# Pharmacological and Behavioral Treatment of Opioid Use Disorder

**DOI:** 10.1176/appi.prcp.20180006

**Published:** 2018-12-05

**Authors:** Mehmet Sofuoglu, Elise E. DeVito, Kathleen M. Carroll

**Affiliations:** ^1^ Yale University School of Medicine Department of Psychiatry; ^2^ VA Connecticut Healthcare System West Haven CT

**Keywords:** opioid, medication‐assisted treatment (MAT), behavioral therapy, methadone, buprenorphine, naltrexone

## Abstract

**Objective:**

Opioid use disorder (OUD) in the United States has surged, with an estimated 2.5 million needing treatment. The aim of this article is to provide a clinical overview of the key pharmacological and behavioral treatments for OUD.

**Methods:**

A nonsystematic review of the literature was conducted to investigate OUD treatments, including their mechanism of action, efficacy, clinical guidelines in the United States, and consideration of frequently occurring comorbid conditions.

**Results:**

Food and Drug Administration (FDA)–approved pharmacotherapies for OUD include methadone, buprenorphine, and naltrexone, each of which has different actions on opioid receptors. Although these medications all show efficacy in some dosages and formulations, barriers to accessibility may be most pronounced for methadone, whereas treatment retention poses greater challenges for naltrexone and, to a lesser extent, buprenorphine. Lofexidine, an α_2_‐adrenergic agonist, has recently been approved by the FDA for treatment of opioid withdrawal symptoms. OUD is commonly treated with medication‐assisted treatment (MAT), which offers pharmacotherapy in the context of counseling and/or behavioral treatments. Behavioral therapies, rarely offered as stand‐alone treatments for OUD, are generally used in the context of MAT, in structured settings or to prevent relapse after detoxification and stabilization. The aim of behavioral interventions is to improve medication compliance and target problems not addressed with medication alone. Individuals with OUD commonly have other comorbid psychiatric and substance use conditions, which are not exclusionary for initiating MAT but should be carefully evaluated and monitored because they may reduce treatment effectiveness.

**Conclusions:**

MAT is the first‐line treatment for patients with OUD and should be provided in combination with behavioral interventions. Treatment retention remains challenging in this population. Future studies should focus on approaches that will serve the complex needs of patients with OUD, including those with comorbid psychiatric and substance use conditions.

**Publisher's Note:** This article is part of a special issue titled “The Opioid Crisis: Filling in the Picture.”

In this article, we review the current pharmacological and behavioral treatments for opioid use disorder (OUD). We have chosen to focus on the clinical aspects of OUD, rather than provide a comprehensive review of the literature. To provide a context for the review, we first provide a brief overview of the opioid epidemic and summarize some key clinical aspects of OUD, including comorbid disorders commonly encountered by individuals with OUD. Next, we review the pharmacological and behavioral treatments for OUD, followed by treatment approaches for OUD in the context of comorbid disorders. We then identify gaps in our knowledge of OUD treatments and future directions for research.

## U.S. Opioid Epidemic

The United States is facing an opioid epidemic, which started in the 1990s and has accelerated during the past decade. In 2016, opioid overdoses were responsible for more than 42,000 deaths (1), and the total number of opioid overdose deaths since 1999 has surpassed 350,000 (2). Since 2013, deaths due to illicitly manufactured fentanyl have increased significantly, and it has been estimated that about half of the opioid overdose deaths involve fentanyl (3). Opioid overdose deaths are mainly due to respiratory depression and frequently occur in the presence of other drugs of abuse, including alcohol, cocaine, and benzodiazepines. In a study of veterans, about half the patients who died from opioid overdose had been coprescribed benzodiazepines, demonstrating the risks associated with combined prescribing of these drugs (4). Drug overdose (led by opioid overdose) has become the leading cause of accidental death in the United States (2), surpassing deaths caused by motor vehicle accidents or gun violence (5). Both heroin and prescription opioids contribute to opioid overdose deaths, with differences according to age. Heroin overdose is more common among adults ages 20–34, and prescription opioid overdose is more common among adults ages 45–59.

Many factors have contributed to the current U.S. opioid epidemic. Perhaps most significant has been the gross underestimation of the addictive potential of opioids for the treatment of noncancer pain. This underestimation was aggressively promoted by the pharmaceutical industry and accepted by the medical and scientific community (6). With the resultant acceptability of opioid use for noncancer pain, an exponential increase in the number of opioid prescriptions has been seen. In 2012, more than 259 million prescriptions were written for opioids, enough to give every U.S. adult his or her own bottle of pills (7). This dramatic increase in opioid prescription has led to a new generation of individuals with OUD. Until the 1990s, first exposure to opioids was typically through heroin and less likely through prescription opioids. Beginning in the 1990s, this pattern started to change, and currently four of five new heroin users initiate opioid use with a prescription opioid, mostly obtained from friends, family, or a dealer (8). It is estimated that in the United States, more than 12 million people use prescription opioids for nonmedical purposes (1). Alarming rates of opioid use have been observed among adolescents, with an estimated 276,000 using prescription opioids for nonmedical use and 21,000 using heroin. Among the estimated 2.5 million people with OUD, it is estimated that 600,000 are abusing heroin and 1.9 million are using prescription opioids (9). This large number of estimated people with OUD creates a challenge for the U.S. medical community regarding optimal screening, diagnosis, and treatment.

## Clinical Characteristics of OUD

OUD is a chronic relapsing disorder. According to the *DSM‐5*, OUD is characterized by a problematic pattern of opioid use and preoccupation with obtaining and taking opioids, as well as using more than intended despite personal, medical, and psychosocial consequences (10). Individuals with OUD typically develop tolerance to opioids and experience withdrawal symptoms upon cessation. The mortality rate of individuals with OUD is 6–20 times greater than mortality rates for the general population (11). Among those who survive, prevalence of stable abstinence from opioid use is less than 30% even after 10 years of follow‐up, and many of those who abstain from opioid use continue to use alcohol and other drugs (11). The medical consequences of OUD include overdose, human immunodeficiency virus (HIV), hepatitis C virus, and other infections contracted through intravenous drug use. The psychosocial burden of OUD includes unemployment, financial problems, homelessness, and legal problems. In fact, OUD was found to have the greatest burden of disease attributable to any illicit drug (12).

## Comorbidities and Risk Factors Associated With OUD

OUD is comorbid with many psychiatric and substance use disorders. In clinical samples of individuals with OUD, rates of current substance use disorder range from 13%–49% for alcohol, 20%–40% for stimulants, 28%–41% for cannabis, and 80%–95% for tobacco (13, 14). Rates of comorbid psychiatric disorders are 28%–35% for major depression, 11% for bipolar disorder, 17%–30% for anxiety disorders, and 10% for posttraumatic stress disorder (PTSD) (13, 14). Although some of these comorbid conditions precede OUD, others emerge after its onset (15). For example, tobacco, alcohol, or cannabis use frequently precedes OUD (16). In contrast, anxiety, chronic pain, or depression may precede or follow development of OUD (17, 18, 19).

It has been estimated that about 23% of people exposed to opioids develop OUD (20), a risk of transition to addiction second only to tobacco (32%) and higher than that for cocaine (17%), alcohol (15%), or cannabis (9%). Risk factors for OUD include genetic as well as psychosocial factors, including history of sexual and/or physical abuse. Evidence suggests that presence of comorbid disorders may increase the likelihood of prescription opioid misuse and OUD (21). In a study of more than 1.2 million opioid prescriptions written for 16‐ to 18‐year‐olds, individuals with pre‐existing psychiatric problems, including anxiety, mood, neurodevelopmental, sleep, and nonopioid substance use disorders, were more likely to be prescribed long‐term (e.g., >90 days) opioids (e.g., for pain) (22). In another study, based on National Survey on Drug Use and Health data of adolescents ages 12–17, major depression was predictive of nonmedical use of prescription opioids and OUD (15). Similar findings have been observed in adult populations. Martins et al., in a study using data from the National Epidemiologic Survey on Alcohol and Related Conditions, showed that preexisting psychiatric disorders, including major depression, bipolar I disorder, panic, and generalized anxiety disorders, were associated with increased risk for nonmedical opioid use (23). In studies of military veterans and civilians, presence of PTSD was predictive of nonmedical prescription opioid use by men and women and of increased risk for OUD in women (24, 25). In sum, these studies highlight the importance of psychiatric comorbidities as risk factors for OUD.

## Current Treatments for OUD

Available treatments for OUD consist of pharmacotherapy and behavioral therapies. The gold standard is medication‐assisted treatment (MAT), wherein pharmacotherapy is combined with some form of counseling or behavioral therapy.

## Pharmacological Treatments for OUD

The FDA has approved methadone, buprenorphine, and naltrexone for treatment of OUD. In addition, the FDA has recently approved lofexidine, an α_2_‐adrenergic agonist similar to clonidine, for treatment of opioid withdrawal. Comprehensive summaries of practice guidelines for MAT are available (26, 27).

### Methadone

#### Mechanisms of action.

Methadone is an opioid agonist. Methadone's effects on euphoria, analgesia, drowsiness, constriction of pupils, nausea, vomiting, constipation, itching, and respiratory depression are mediated mainly by its mu‐opioid receptor (MOR) agonist effects (28). The goals of methadone treatment are suppression of opioid withdrawal symptoms and opioid craving and cessation of illicit opioid use. At higher doses (i.e., >100 mg/day), methadone induces opioid tolerance, resulting in reduced reinforcement from other opioids (such as heroin) through the development of cross tolerance (29).

#### Efficacy.

Methadone has been the gold standard pharmacotherapy for opioid maintenance and detoxification since its introduction in the 1960s. Methadone maintenance treatment (MMT) is effective in reducing illicit opioid use, preventing HIV infection, and improving retention in treatment (30). In a Cochrane review comparing methadone versus no‐methadone treatment, Mattick et al. found that methadone reduced heroin use but not mortality or criminal activity (31). Sordo et al., in another meta‐analysis of studies examining mortality rates during and after methadone treatment, found that retention in treatment was associated with reduced all‐cause mortality (11.3 and 36.1 per 1,000 person‐years in and out of methadone treatment, respectively) (32). The first four weeks after leaving treatment were especially associated with high mortality, possibly due to loss of opioid tolerance and return to opioid use.

#### Prescribing guidelines and dosage.

In the United States, prescription of methadone for maintenance treatment of OUD is permissible only in federally licensed opioid treatment programs. As demonstrated by a study conducted in France, however, methadone can be used effectively in primary care settings for the treatment of OUD (33). Methadone is well absorbed following oral intake. Its effects start within 15–45 minutes, and peak plasma levels are reached within 2.5–4 hours (29). Methadone suppresses opioid withdrawal symptoms with once‐a‐day dosing because of its long half‐life (24–36 hours). Methadone's analgesic effects, however, last only 4–8 hours, necessitating multiple daily dosing if prescribed for pain management. With greater use of methadone for pain management in individuals without OUD beginning in the early 2000s, methadone‐related overdose deaths increased, responsible for 5,500 U.S. deaths in 2007 (34). With greater efforts to educate physicians in the proper use of methadone, methadone‐related overdose deaths in the United States have started to decline during the past decade (i.e., 39% decline from 2007 to 2014) (35).

For maintenance treatment of opioid dependence, the usual starting dose of methadone is 20–30 mg, with 5‐ to 10‐mg increases every other day as tolerated. With repeated dosing, methadone can accumulate in the body, increasing risk for respiratory depression and sedation and requiring careful and slow titration in treatment initiation (36). It may take a couple weeks to reach a daily methadone dose that is effective for OUD. Treatment initiation carries a high risk of treatment dropout and for opioid overdose if titration is too rapid (37).

The usual maintenance dose of methadone administered in MMT programs ranges from 30 to 100 mg. Methadone doses of 60 mg/day or higher are more effective than doses less than 60 mg/day in reducing illicit opioid use and improving treatment retention (38, 39). Thus, although 60 mg/day of methadone is recommended as the minimum, doses up to 100 mg/day should be considered, especially for patients with ongoing opioid use (40). Methadone doses greater than 100 mg/day may be indicated, especially for those receiving medications that speed the breakdown (i.e., increase the metabolism) of methadone, such as some medications used to treat HIV infection. Although lower doses may be sufficient to suppress opioid withdrawal symptoms and opioid craving, at higher doses (100 mg or more/day) methadone also induces a high level of opioid tolerance, resulting in reduced reinforcement from other opioids (such as heroin) by development of cross‐tolerance (29).

Although the optimum duration of MMT is controversial, longer treatment is associated with better outcomes, and discontinuation of methadone treatment is associated with high risk of relapse to illicit opioid use and death by overdose (41). In a benchmark study, treatment retention and reduction of opioid use outcomes were better in the group randomized to MMT (14 months of MMT followed by two months of detoxication) versus the group randomized to 180‐day methadone‐assisted detoxification (six months of gradual detoxification off methadone, followed by continued access to psychosocial platform therapies for the remaining eight months); both treatment conditions were offered a psychosocial platform therapy across the entire trial (42). Patients may require continuous methadone treatment for years and perhaps for life, similar to treatment for other chronic medical disorders (43, 44).

#### Adverse effects.

Adverse effects of methadone are similar to those of other opioids and include nausea, vomiting, constipation, excessive sweating, decreased libido, reduced testosterone in men and luteinizing hormone in women, reduced pain threshold (hyperalgesia), and impaired cognitive function (36, 45, 46). Methadone also can prolong the cardiac QT interval, increasing risk for life‐threatening arrhythmias. It is recommended that an electrocardiogram be obtained before treatment initiation or dose increases (47). With chronic treatment, tolerance develops to most of methadone's MOR‐mediated pharmacological effects (e.g., euphoria, analgesia, drowsiness, constriction of pupils, nausea, vomiting, constipation, itching, and respiratory depression) (28). However, tolerance dissipates within a couple days of abstinence, resulting in increased risk of overdose following relapse and resumption of treatment at the individual's usual dosage. Reduction of the individual's usual dose is prudent following missed doses of methadone in patients participating in MMT.

Adherence to MMT varies significantly. Predictors of poor adherence include being female, single, never married, or homeless; using other drugs, including alcohol; having a positive drug screen at the time of admission; and having comorbid psychiatric disorders (48).

### Buprenorphine

#### Mechanisms of action.

Buprenorphine is a partial MOR agonist and a weak kappa‐opioid receptor (KOR) antagonist (49). In clinically used doses, buprenorphine acts like a typical MOR agonist, such as morphine or methadone. At higher doses, however, buprenorphine's MOR agonist effects reach a plateau and its KOR antagonist effects begin to emerge. This ceiling effect of buprenorphine reduces its abuse potential and risk of overdose even at high intravenous doses (50). Further, its slow dissociation from opioid receptors allows flexible dosing that can range from several times a day to three times per week (51).

#### Efficacy.

Buprenorphine either alone or in combination with naloxone is safe and effective for treatment of OUD (50). In a meta‐analysis, Nielsen et al. found buprenorphine similarly effective to methadone in reducing opioid use (52).

#### Prescribing guidelines and dosage.

Since its approval in 2002, buprenorphine has been increasingly used in the United States for treatment of OUD (53). Buprenorphine is unique in that it allows physicians, nurse practitioners, and physician assistants to provide office‐based opioid maintenance treatment after completing training and receiving a Drug Enforcement Administration waiver (54). Each qualified practitioner may treat up to 30 office‐based buprenorphine patients in the first year and 100 patients in the second year, up to maximum of 275 patients at a time for qualified providers. Office‐based buprenorphine treatment has greatly increased the capacity for OUD treatment in the United States.

Buprenorphine, formulated as a sublingual tablet or film, is available as a monotherapy or in a combination tablet (Bup‐Nx) containing buprenorphine and naloxone in a 4:1 ratio (50). Because naloxone is poorly absorbed when taken orally or sublingually, its effects are negligible when the medication is taken as directed, and it is included solely as a deterrent against manipulation of the product and administration through any other than the intended oral or sublingual routes. Injection of the combination tablet in dependent individuals leads to naloxone‐precipitated opioid withdrawal, which deters diversion of this product to injection drug use (55). Buprenorphine monotherapy is administered in the clinic, whereas the combination tablet is appropriate for take‐home use (56, 57). Buprenorphine monotherapy is preferred for pregnant women with OUD (58). Buprenorphine monotherapy is also available in injectable and subdermal implant routes, which were recently approved by the FDA for treatment of OUD. The injectable form can be given once a month after the patient is stabilized on buprenorphine for at least one week. The implant provides a steady level of buprenorphine for up to 6 months for those maintained on a stable dosage of buprenorphine of ≤8 mg/day.

Following sublingual administration, peak levels of buprenorphine are reached within 30–60 minutes. The plasma elimination half‐life of buprenorphine is 37 hours (55). Because of its partial agonist actions at MOR, buprenorphine may precipitate withdrawal if initiation of treatment is not managed appropriately. To minimize this possibility, patients should be asked not to use opioids and to wait for emergence of mild withdrawal symptoms before initiating buprenorphine treatment. The recommended starting dose is 2–4 mg of buprenorphine, which may be followed in 3–4 hours with an additional dose of up to 4 mg based on the patient's clinical response. On the second day, the dose of buprenorphine may be increased to 12–16 mg, and the stabilization dose, ranging from 8 to 24 mg/day, may be reached within the first week.

After a stable dosage is reached, the patient may be switched to three times weekly doses (e.g., Monday, Wednesday, and Friday). This schedule may require further dosage adjustment. For example, an 8‐mg daily dose of buprenorphine may be switched to 16 mg, 16 mg, and 24 mg for Monday, Wednesday, and Friday, respectively. Similar to methadone, longer treatment retention with buprenorphine is associated with better outcomes, and treatment discontinuation leads to high rates of relapse (37).

#### Adverse effects.

Adverse effects of buprenorphine are similar to those of methadone and include dry mouth, nausea, vomiting, constipation, dizziness, sedation, headache, and excessive sweating. Buprenorphine is not associated with prolonged QT interval and has lower overdose risk than methadone (40).

### Naltrexone

#### Mechanisms of action.

Naltrexone is a competitive antagonist of opioid receptors; of the opioid receptors, naltrexone has the highest affinity to MOR and less affinity to delta opioid receptors and KOR (59). Naltrexone in clinically used doses effectively blocks the subjective, reinforcing, and physiological effects of MOR agonists such as hydromorphone (60).

#### Efficacy.

Oral naltrexone was approved by the FDA for the treatment of OUD in 1984 (61). Based on its pharmacological properties and favorable adverse effect profile, naltrexone theoretically would appear to be an almost perfect medication for relapse prevention of OUD, with unreinforced opioid use (i.e., blocking of opioid's rewarding effects) leading to extinction of the behavior. Unfortunately, in clinical practice, the difficulty of initiation, poor compliance and high dropout rates have limited the usefulness of oral naltrexone as a treatment for OUD. In fact, Minozzi et al., in a Cochrane meta‐analysis, found no significant differences between oral naltrexone and placebo for treatment retention and opioid use (61).

To improve the compliance and effectiveness of naltrexone, two long‐acting depot formulations for naltrexone have been developed: an extended‐release intramuscular injection (XR‐NTX) and a subdermal implant (SBM NTX) (62). XR‐NTX is FDA approved for alcohol use disorders and OUD. The subdermal implant has not yet received FDA approval.

#### Prescribing guidelines and dosage.

Oral naltrexone can be delivered in a once‐daily dose, due to the relatively long half‐life of its active metabolite (naltrexone and its primary active metabolite, 6‐beta‐naltrexol, have plasma half‐lives of four and 13 hours, respectively). XR‐NTX, which contains 380 mg of naltrexone, is given monthly via intramuscular injection. To minimize precipitation of opioid withdrawal, oral or XR‐NTX treatment should not be initiated until the patient is opioid free for 7–10 days, as recommended by the manufacturer. Induction of opioid abstinence, with prolonged symptoms of opioid withdrawal during this transition, however, increases risk of treatment dropout and relapse to nonmedical opioid use.

Currently, there is no established abstinence and naltrexone induction protocol for XR‐NTX. Commonly used approaches include a brief tapering of buprenorphine (7–14 days) followed by 7–10 days of washout (63). However, this approach leads to a high dropout rate and relapse to nonmedical opioid use because of the severity of opioid withdrawal symptoms. To better manage the withdrawal symptoms during the washout period, other protocols have been developed. One alternative is to have 1–2 days of washout after the last buprenorphine dose and a gradual titration of very low doses of oral naltrexone (1–3 mg) during the next 3–5 days (64). Patients also may be provided a standing order for doses of medications (e.g., clonidine and clonazepam) to alleviate opioid withdrawal symptoms (64). Another alternative includes buprenorphine tapering combined with very low doses of oral naltrexone (0.25–1 mg) for 2–3 days. This tapering is followed by gradual titration to full blocking doses of naltrexone (>25 mg) (65). By following these procedures, 50%–70% of outpatients were able to initiate treatment with XR‐NTX (63). Jarvis et al., in a recent meta‐analysis (66), found that XR‐NTX induction success was lower in studies that included individuals taking opioids when they were assigned to treatment and who required detoxification before they could initiate XR‐NTX compared with studies of individuals who had already completed detoxification from opioids at the time they were assigned to XR‐NTX (63% vs. 85%), suggesting the delay in initiation of XR‐NTX or other aspects of the process of detoxification prior to initiation of XR‐NTX provides a barrier to treatment. Adherence was higher in prospective studies than in retrospective studies of medical records taken from routine clinical care (6‐month rates: 46.7% vs. 10.5%). Jarvis et al. concluded that two main factors that limit the clinical utility of XR‐NTX are the difficulty of initiating the treatment and the high dropout rate among those initiating the treatment (66).

#### Adverse effects.

Naltrexone's adverse effects include insomnia, injection site pain (injectable form), nausea, headaches, hypertension, nasopharyngitis, influenza, and clinically insignificant transaminase elevation. Whether naltrexone induces depression or anhedonia is an area for research (67, 68).

## Comparison of FDA‐Approved OUD Pharmacotherapies

### Methadone, Buprenorphine, and Naltrexone for MAT

Several recent trials have compared buprenorphine and methadone for the treatment of OUD (69, 70). In general, the results of these trials indicate significantly improved patient retention in methadone compared to buprenorphine maintenance, with similar outcomes on opioid use (71). Consistent with the better retention with methadone than buprenorphine in clinical trials, researchers conducting observational studies have reported that patients drop out of buprenorphine treatment more rapidly than methadone treatment (72, 73). Several possibilities have been raised to explain the better retention with methadone than with buprenorphine. First, methadone programs in the United States provide greater structure and support than office‐based buprenorphine treatment, which may contribute to the better retention with methadone. The second possibility relates to differences between the pharmacological actions of these medications on the MOR. Methadone, with its high activity at the MOR, is highly reinforcing and induces severe withdrawal upon cessation. Further, the opioid blockage induced by methadone, especially when given in high doses, has been linked to better outcomes (38, 39). In contrast, due to its partial agonist effects on MOR and antagonist effects on KOR, buprenorphine is less reinforcing and induces a milder withdrawal, making it easier for patients to drop out (74).

In a recent multisite clinical trial comparing intramuscular extended release naltrexone (XR‐NTX) to the Bup‐Nx combination tablet, where treatment was started in inpatient settings to try to circumvent the difficulties with the induction phase for naltrexone (75), Lee et al. found that induction failures were higher for patients randomized to XR‐NTX (28% vs. 6% for patients randomized to Bup‐Nx); relapse rates also were significantly higher with XR‐NTX than with Bup‐Nx (65% vs. 57%); however, when evaluating data only from those successfully inducted, rates of relapse and retention were roughly comparable, as were safety profiles. In both groups, however, 24‐week retention rates were modest (47% for XR‐NTX and 43% for Bup‐Nx) (75).

In a Norwegian study, prisoners with OUD who received either open‐label methadone or a naltrexone subdermal implant (which is not yet FDA approved) prior to release from prison showed equivalent decreases in opioid and benzodiazepine use and criminality outcomes in the six months following release compared with their behavior before imprisonment, wherein self‐reported criminality within each timeframe was measured with the Addiction Severity Index (76).

### α_2_‐Adrenergic Agonists: Clonidine and Lofexidine for OWS

Clonidine and lofexidine reduce adrenergic output by stimulating the inhibitory α_2_‐adrenergic receptors. Clonidine is FDA‐approved for treatment of essential hypertension and attention‐deficit hyperactivity disorder. It is commonly used off label to alleviate opioid withdrawal symptoms (OWS). However, a dosage schedule for the use of clonidine as a treatment for OWS has not been established. Clonidine's adverse effects include hypotension, sedation, and dizziness. The typical dose varies from 0.3 to 0.6 mg, given in multiple divided doses as needed. Beyond its use for OWS, clonidine may reduce opioid use by attenuating stress and cue‐induced craving. In a randomized clinical trial with opioid users maintained on buprenorphine, investigators found that clonidine, compared to placebo, significantly reduced stress or cue‐induced craving for heroin and prevented relapse to heroin use following abstinence (77). These findings suggest that clonidine may improve MAT efficacy by reducing stress and drug cue reactivity.

Lofexidine, which has been used in the United Kingdom for OWS since 1992, has recently been approved by the FDA for treatment of OWS. Lofexidine's pharmacological effects are similar to those of clonidine but are less likely to cause hypotension. In clinical trials of lofexidine for OWS, treatment duration has ranged from 1 to 3 weeks (78). The initial dosage of lofexidine is 0.2 mg twice daily, which can be increased by 0.2–0.4 mg/day until the final dosage (1.6–3.2 mg/day) is reached. In systematic reviews, lofexidine was found to be more effective than placebo in attenuating OWS (78). Whether or not lofexidine enhances the efficacy of MAT in reducing opioid use, as does clonidine, has not been examined.

## Behavioral Treatments for OUD

In general, behavioral therapies, when delivered alone, have limited efficacy in addressing the complex symptomatology and physical aspects of OUD. Thus, behavioral therapies for OUD have been delivered in the context of structured approaches (e.g., residential programs), after completion of detoxification and stabilization to prevent relapse (79, 80) or, most effectively, in the context of MAT (i.e., in combination with an approved medication: methadone, buprenorphine, or naltrexone) (81, 82). When delivered in the context of MAT, the roles of behavioral therapies have been to improve adherence to the medication, address aspects of the disorder not addressed by pharmacotherapy, and address specific weaknesses of the pharmacotherapy. Thus, we will focus this review of behavioral therapies on their delivery in conjunction with methadone, buprenorphine, or naltrexone.

### Behavioral Therapy With Methadone

The key early study demonstrating the value of behavioral therapy within methadone maintenance was conducted by Woody and colleagues (83). In that study, 110 individuals entering methadone maintenance were randomly assigned to one of three treatments: drug counseling alone, drug counseling plus supportive‐expressive psychotherapy (a short‐term dynamic approach), or drug counseling plus cognitive psychotherapy (a structured cognitive approach) (83). At the 6‐month follow‐up, the supportive‐expressive and cognitive psychotherapy groups did not differ significantly from each other on most outcome measures. Individuals in either of the groups receiving professional psychotherapy showed greater improvement in outcomes than those receiving drug counseling alone (83). Gains made by those who received psychotherapy were sustained during a 12‐month follow‐up, whereas those who received drug counseling alone showed some attrition of gains (84).

Several studies have evaluated the use of contingency management (CM) approaches to reduce illicit drug use by people with addiction and receiving MMT. In these studies, a reinforcer (reward) is provided to patients who demonstrate specified target behaviors, such as providing drug‐free urine specimens, accomplishing specific treatment goals, or attending treatment sessions. For example, a positive contingency may consist of offering methadone take‐home privileges (i.e., the reinforcer/reward) contingent on reduced illicit drug use (i.e., the behavior being reinforced). This approach capitalizes on an inexpensive reinforcer that is potentially available in all methadone maintenance programs. Stitzer and colleagues demonstrated that such positive contingencies had dramatic effects on reduction of illicit opioid use (85, 86, 87). Since then, numerous trials have documented the efficacy of a variety of CM systems (e.g., receiving vouchers, chances to win inexpensive prizes, access to paid work) in the context of methadone maintenance, with particularly strong effects when reduced comorbid cocaine use is the behavior being reinforced (88, 89). Despite the strength of the evidence supporting CM to improve treatment outcomes in the context of methadone maintenance, CM has not achieved broad clinical use, primarily because of the cost of the rewards, although lower‐cost efficacious CM systems have been developed (90, 91), and because of the tendency for the effects to weaken after the rewards have been terminated posttreatment (92).

Although the type and quality of behavioral therapies vary widely in differing methadone maintenance programs, extensive literature indicates large differences in retention and outcome that vary with the intensity and quality of the services provided (93). For example, in the landmark study on the effects of behavioral interventions and services in MMT, McLellan and colleagues randomly assigned 92 opioid‐dependent men who were stabilized on methadone to either no additional services, standard services plus counseling, or enhanced services, which provided counseling plus individually tailored, on‐site medical, psychiatric, employment, and family therapy services (94). Although some individuals did well as part of the no‐additional‐services group, 69% had to be protectively transferred out of the study due to “unremitting use of opiates or cocaine, or medical/psychiatric emergencies” (94). Outcomes differed significantly across the study groups, with the best outcomes in the enhanced services group and the poorest ones in the no‐additional‐services group. This study and others have led to a general, although not universal (95, 96, 97), consensus that behavioral intervention is a key component of successful methadone treatment programs (98).

### Behavioral Therapy With Buprenorphine

The role of behavioral therapies in the context of buprenorphine maintenance also has been controversial, and findings have varied based on the nature of the “treatment as usual” comparator condition. The value of behavioral therapy as an adjunct to buprenorphine was called into question by the results of four trials that indicated that, in the context of medical management (weekly meetings with a physician and urine monitoring), the provision of additional behavioral therapy or counseling provided no significant additional benefit on treatment retention or opioid use outcomes (99, 100, 101, 102). However, the “treatment as usual” condition of those studies—namely medical management—may be difficult to implement or sustain in clinical practice and, therefore, is not representative of standard treatment offered in most clinics (103). In contrast, several studies that did not use intensive medical management as a platform treatment have shown that specific well validated behavioral therapies, particularly contingency management and brief Web‐based interventions, are associated with significantly improved retention and outcomes in buprenorphine treatment (104, 105, 106, 107).

The controversy regarding the importance and role of behavioral therapies in the context of buprenorphine maintenance may reflect, in part, the differing perspectives of the most effective approaches to improving treatment impact. Individuals focusing on harm reduction emphasize the demonstrated benefits of buprenorphine treatment on reducing mortality (71) and prioritize the goal of broadening availability and accessibility to U.S. buprenorphine treatment programs. From this harm‐reduction perspective, any added complexity or cost of treatment (e.g., adjunct behavioral therapies) may be viewed as interfering with the goal of expanding access to MAT, which, given the current opioid crisis, is the paramount public health concern. In contrast, those who focus on improving outcomes see the limitations of buprenorphine maintenance as commonly delivered: six‐month retention rates seldom exceeding 50%, dropout associated with high risk of relapse and mortality (37, 108, 109), and poor clinical outcomes [100]. Because, the latter perspective prioritizes the goal of optimizing treatment retention and outcomes, adjunct behavioral therapies are viewed as valuable tools.

An ideal approach likely balances these perspectives: increasing access to meet demands while also considering ways to optimize treatment engagement and efficacy. Thus, some important questions facing the field are what types of patients fare well with buprenorphine maintenance without additional behavioral therapy and what models of behavioral intervention are both practical and helpful in the context of office‐based buprenorphine treatment (82)? While research addressing these issues continues, providers might consider a stepped‐care model for patients on buprenorphine, in which the intensity of visits and counseling is tailored to patient response, as assessed by buprenorphine adherence and regular urine toxicology screening (98).

### Behavioral Therapy With Naltrexone

As described above, oral naltrexone, despite its theoretical advantages, has not fulfilled its promise. This result is largely due to problems with retention, particularly during the induction phase, in which an average of 40% of patients drop out during the first month of treatment and 60% drop out by three months (110). CM programs specifically rewarding adherence to naltrexone and reducing illicit drug use have been consistently effective (111, 112, 113, 114) but have been challenging to move into broad clinical practice (115).

Depot formulations of naltrexone have shown benefit in retention over oral naltrexone, but retention is higher in populations in which there are clear consequences for nonadherence (e.g., in patients whose professional licensure is contingent on compliance and in criminal justice populations) (66). In general, in clinical samples, six‐month retention is low, with the modal number of injections seldom exceeding one or two (66). Because depot formulations have only recently been approved, limited research exists on specific behavioral strategies to enhance retention and outcomes in these patient populations; early studies again suggest the promise of CM approaches (116). Poor retention even in tightly controlled studies, coupled with the high cost of detoxification and of the drug itself, indicate more effective strategies for improving naltrexone retention and outcomes are needed.

## Interim Treatment of OUD

Given the high demand and limited capacity for MAT, individuals with OUD can remain on the waiting list for months before initiating MAT. To minimize the risk for mortality and morbidity associated with untreated OUD, the FDA has approved interim treatment which includes daily buprenorphine or methadone and emergency counseling (117). Several studies have found interim treatment more effective than waitlist in reducing opioid use and increasing retention and entry into long‐term treatment (118). Further studies are needed to examine the efficacy of interim treatment and to identify the subgroup of patients most likely to benefit from such treatment.

## Treatment of OUD With Psychiatric Comorbidities

Despite their common occurrence, comorbid psychiatric conditions associated with OUD have received limited attention, especially regarding their impact on the clinical management of OUD. For individuals with OUD, presence of psychiatric comorbid conditions often is associated with worse outcomes (119). The high rates of polypharmacy encountered in patients with comorbid psychiatric conditions may reduce compliance with MAT and may increase the risk of adverse drug interaction (120).

Symptoms of depression or anxiety are commonly observed at treatment entry in patients with OUD. Because most of these symptoms subside within a month after the initiation and regular use of MAT, specific behavioral or pharmacological interventions should be considered only in the absence of substantial improvement (119). However, if a pre‐existing depression or anxiety disorder is well established, specific treatment can be initiated simultaneously with MAT. Among pharmacological treatments, tricyclic antidepressants but not SSRIs have shown some efficacy for the treatment of depressive and anxiety symptoms in patients receiving MAT (119). It is important to note that tricyclic antidepressants are associated with adverse effects, including sedation, anticholinergic effects, and seizures that need careful monitoring. The few available studies also suggest that differing behavioral therapies, including cognitive‐behavioral therapy, acceptance and commitment therapy, and relational psychotherapy, are effective in reducing depressive as well as anxiety symptoms and should be considered as treatment options (119).

A high degree of comorbidity also has been observed between PTSD and OUD (121). A hyperadrenergic state may be a common mechanism for both OWS and PTSD, and clinically it may be difficult to separate OWS from the hypervigilance and increased arousal of PTSD. In patients with comorbid PTSD and OUD, methadone treatment improves OUD outcomes with minimal effects on PTSD symptoms (121). The efficacy of buprenorphine or naltrexone has not been systematically examined among patients with PTSD and OUD. There is a need for integrated treatment that will target both PTSD and OUD.

Patients with OUD, especially those taking MAT, commonly use other drugs of abuse, including alcohol, benzodiazepines, cocaine, and tobacco. In general, MAT should not be withheld from individuals because of other drug use. However, patients should be assessed and monitored for their nonopioid drug use during treatment because these drugs may adversely affect treatment outcomes. Approximately one third of individuals on methadone maintenance treatment report problematic alcohol use or alcohol use disorder (122), a disorder that is predictive of poor treatment outcome among patients taking MAT. Repeated alcohol intoxication poses a safety risk, especially with opioid agonist treatments (122). Monitoring of alcohol intake via Breathalyzer and addressing ongoing alcohol use early are recommended. Similar to alcohol use disorder, benzodiazepine use increases the risk of opioid overdose and is predictive of poor treatment outcomes in patients taking MAT (123). Preventing patients from doctor shopping to elicit benzodiazepine prescriptions, switching patients to a long‐acting benzodiazepine (e.g., clonazepam), and initiating slow benzodiazepine tapering are recommended (124). Cocaine use is also common, especially by individuals with OUD who are maintained on methadone (125). Combining intravenous cocaine and heroin (i.e., speedballing) is associated with high mortality (126). OUD is highly comorbid with tobacco use disorder, with more than 95% of MAT patients reporting current cigarette smoking (127). Although evidence‐based pharmacological and behavioral treatments for tobacco use disorder are less effective in those with OUD (128), efforts should be made to help individuals to quit cigarette smoking.

## Conclusions

OUD continues to be major public health problem in the United States. MAT is the first‐line treatment for OUD. Overall, treatment retention is challenging in this patient population, which undermines treatment efficacy. MAT should be provided in combination with behavioral interventions to improve treatment outcomes. Future studies should focus on treatment approaches that will better serve the complex needs of patients with OUD, including those with comorbid psychiatric and substance use conditions.
